# Diabetic health literacy and its association with glycemic control among adult patients with type 2 diabetes mellitus attending the outpatient clinic of a university hospital in Ethiopia

**DOI:** 10.1371/journal.pone.0231291

**Published:** 2020-04-08

**Authors:** Yonas Getaye Tefera, Begashaw Melaku Gebresillassie, Yohannes Kelifa Emiru, Ruth Yilma, Firdos Hafiz, Henok Akalu, Asnakew Achaw Ayele

**Affiliations:** 1 Department of Clinical Pharmacy, School of Pharmacy, College of Medicine and Health Science, University of Gondar, Gondar, Ethiopia; 2 Department of Pharmacognosy, School of Pharmacy, College of Medicine and Health Science, University of Gondar, Gondar, Ethiopia; Chinese Academy of Medical Sciences and Peking Union Medical College, CHINA

## Abstract

**Background:**

Despite how much effect of low health literacy is on diabetic treatment cannot be accurate, it has an impact on controlling blood glucose level. Less is known about diabetic health literacy in Ethiopian diabetic patients which can affect patient medication adherence, self-care, and glycemic control.

**Objective:**

This study was aimed to assess the diabetic health literacy level and its association with glycemic control among adult patients with type 2 diabetes mellitus attending the outpatient clinic of University of Gondar Comprehensive Specialized Hospital (UOGCSH): Northwest Ethiopia.

**Methods:**

A cross-sectional study was employed at the outpatient clinic of the University of Gondar Comprehensive Specialized Hospital from May, 1 –May 30, 2019. The comprehensive 15-items diabetic health literacy questions with a 5-point Likert scale used to measure diabetic health literacy. The mean score calculated and switched to the percentage (5 points as 100%) to determine the level of diabetic health literacy. Morisky Green Levine Scale 4 item adherence assessment tool was used to assess the diabetic patient's level of adherence. Binary and multivariable logistic regression analysis was used to assess the association between sociodemographic, clinical variables, diabetic-related literacy, and glycemic control. Independent samples t-test and One-way ANOVA test was employed to compare the mean literacy score difference in different groups.

**Result:**

400 respondents were included in the study. Of all the respondents, 17.3%, 26.3%, and 56.5% had low, medium and high diabetic-related health literacy, respectively. The proportions of patients with low, medium and high adherence to medication were 9.8%, 56.3%, and 34% respectively. Patients with high diabetes literacy are 1.85 times more likely to achieve target glycemic control than lower diabetic literacy patients with 95% CI Adjusted Odds Ratio (AOR). 1.85(1.09–3.40). While patients with good adherence 1.61 times more likely to achieve target glycemic control than patients with low adherence; 95% CI AOR 1.61(1.04–4.79). Diabetic patients with morbidity have 67% less likelihood to achieve the target glycemic control; 95% CI AOR 0.33(0.15–0.73).

**Conclusion:**

Adequate diabetic health literacy and better glycemic control are highly correlated. Adjusting all variables; younger age, high diabetic health literacy and good adherence are associated with achieving the target glycemic control.

## Introduction

Diabetes mellitus (DM) is a serious global public health problem ranked as the seventh leading cause of death. In 2016, the World Health Organization (WHO) estimated 422 million adults were living with diabetes. The International Diabetic Federation also predicted the number of diabetic patients will rise to 642 million by 2040 [1, 2]. The estimated prevalence of DM in the adult population of Ethiopia is 1.9% [3–5]. Diabetic patients are at high risk for several chronic complication, including end-stage renal disease, blindness, and amputations. Poor control of intermediate risk factors (e.g., glycemic control, cholesterol levels, and blood pressure) plays a significant role in determining the risk [6]. Among these intermediary factors, glycemic control is a crucial anticipator of several diabetic complications [7, 8].

Health literacy has become increasingly crucial for social, economic and health development. If there is an improvement in the health literacy of a community, it will be good to improve health services and disease control [9]. WHO conceptualized health literacy as “the cognitive and social skills which determine the motivation and ability of individuals to gain access, understand, and use information in ways that promote and maintain good health” [10]. Diabetic related health literacy is the extent to which patients with diabetes have the required skills and abilities to seek, understand, analyze, communicate, and enumerate diabetes-related information both in the healthcare environment and daily lives for treating and self-managing their condition [2].

Patients with diabetes require to execute continuing wide self-management protocol including self-regulation of diet, control of plasma glucose levels, physical exercise, medication management (prescribed doses, frequency of administration, and appropriate timing), foot care, and stress management. These self-management methods play a key role in the effective treatment protocol of diabetes. However, patients with low health literacy experience some trouble in realizing health-related information and get difficulties to express their status to health care providers, resulting in poor self-management. Therefore, It became visible as health literacy is a relevant determinant of self-management in diabetes [2]. Inadequate health literacy is a major obstacle to diabetic care or education for patients living with diabetes [11]. Low health literacy in type- 2 diabetic patients associated with failure in managing their plasma glucose level. This was as a result of diabetic patients who has inadequate health literacy usually faced difficulty in their medication prescriptions, reading drug labels, interpreting blood glucose test result or other information received from the health center. Despite how much effect of low health literacy is on diabetic treatment cannot be accurate, many studies have proved that low health literacy has an impact on controlling blood glucose level. Low health literacy can affect self-management and engagement in health promotion and thus be a predisposing factor for poor glycemic control [12].

A capacity to obtain, process, and understand basic health-related information and services that are important to make suitable health decisions has been conceptualized to be a required non-clinical factor that can significantly diminish the risk of adverse health consequences in diabetes [13, 14]. Assessment of health literacy is therefore necessary for providing baseline evidence to design programs and strategies for effective health education [15]. Especially such evidence is highly relevant in countries like Ethiopia where there is a high level of illiteracy rate. Despite the formal education coverage is increasing in Ethiopia, the population literacy status is still low with a total adult literacy rate of 36%. The growing literacy level in the country is still a major marker of influencing the health-seeking behavior of the population such as the use of modern health care service [16]. Less is known about diabetic health literacy in Ethiopian patients which might affect patient medication adherence, self-care and predicting factors for glycemic control. Therefore, this study aimed to assess the diabetic-related Health literacy level and its association with glycemic control among patients with type 2 Diabetes Mellitus attending the outpatient clinic at the University of Gondar Comprehensive specialized hospital in Northwest Ethiopia.

## Methods

### Study design, study area, and period

Institutional based prospective cross-sectional study was conducted among adult type 2 DM patients in UOGCSH from May 1, 2019, to May 30, 2019. It is the largest healthcare facility in North West Ethiopia and estimated to have the highest number of diabetic patients in ambulatory care follow up. With this regard, the chronic ambulatory care clinic is anticipated to serve 6000–7000 diabetic patients living in northwest Ethiopia.

### Inclusion and exclusion criteria

All type 2 DM patients with the age of 18 years and above who visited the clinic for follow-up, at least 6 months on diabetic therapies, and patients not referred for admission for inpatient and emergency care treatment were included for this study. While patients with Gestational Diabetes Mellitus, a mental disorder with cognitive impairment and patients with visual and communication impairment might be difficult to assess their health literacy either in interviews or questionnaire administration were excluded.

### Sample size determination and sampling technique

The sample size was determined using a single proportion formula for a population

$${n=(\frac{Z}{2})}^{2}\frac{P(1-P)}{W^{2}}$$

n = desired sample size, when the study population > 10,000

Z is the standard normal distribution set as 1.96, which corresponds to a 95% confidence. interval

P = Proportion in the target population estimated to have adequate health literacy. There is no reasonable estimate since no prior study has been conducted in the Ethiopian setting, so we used 50% (i.e. 0.5) to maximize the sample size.

1-P = proportion in the target population not having adequate health literacy

w = degree of accuracy required (set at 0.05 a marginal error)

Based on this

n = (1.96^2^)*(0.5)*(0.5)/0.05^2^

n = 384. But the total Diabetic population attending the outpatient clinic was estimated between (N) = 6000–7000 (<10,000). Therefore, we used the correction formula to determine the final sample size.

**n**_**f**_ = $n/(1+\frac{n}{N})$

**n**_**f** =_ the final sample size

**n**_**f =**_ 384 / (1 + 384/7000) = 364

Considering a 10% non-response rate, the final sample size was 400The simple random sampling technique used to select study participants who came for follow-up every Tuesday and Friday at the out-patient clinic. 50 diabetic patients were selected from the daily medical chart registration randomly every Tuesday and Friday during the one-month period. Then patients interviewed for diabetic health literacy questions and relevant clinical information is taken from their medical chart.

### Data collection tool and methods

The comprehensive 15-item diabetic health literacy scale was used to assess the patient’s diabetic health literacy level. The tool measures comprehensive aspects of informational, numeracy, and communicative health literacy relevant to diabetes [10]. Morisky Green Levine Scale 4 item adherence assessment tool was used to assess the diabetic patient's level of adherence [17] [18]. A structured questionnaire which comprised of items focusing on socio-demography, social drug use history, diabetic-related clinical information, 15 diabetic health literacy questions with 5 point Likert scale which mean score calculated and switched to the percentage (5 points as 100%) to determine the level of diabetic health literacy and 4 item adherence questions were used to measure patient adherence behaviors.

Data were collected by three graduating class pharmacy students. Data was collected by both interviewing and self-administration depending on the patient’s educational status.

### Data quality control measures

The questions were translated into Amharic and back-translated to English to conform its original meaning and to maintain unbiased response. The filled questionnaire was checked daily for completeness. The questionnaire was evaluated for its content and modification was done for some literacy questions by adding diabetic medication storage questions specific for our society to the diabetic health literacy questionnaire. The diabetic health literacy scale too was checked for its reliability and showed good internal consistency with Cronbach’s alpha coefficient of 0.928.

### Data entry and statistical analysis

Data were coded, entered and analyzed using SPSS version 24 software. Socio-demographic characteristics, medication, and disease-related information were described by descriptive statistics using frequencies, percentage, mean and standard deviation. Binary and multivariable logistic regression analysis was used to assess the association among sociodemographic variables, clinical information, level of adherence, diabetic-related health literacy level, and glycemic control. Variables included in the adjusted regression model of glycemic control were variables with a p-value of less than 0.3 in the crude ratio regression analysis. Independent samples t-test and One-way ANOVA test was employed to compare the mean literacy score difference among different groups. A chi-square test was used to assess the association between diabetic health literacy level and adherence level of patients. Confidence Interval of 95% and P-value less than 0.05 was used as a cut-point for statistical significance.

### Ethical consideration

Ethical approval was obtained from the ethical review committee of the school of pharmacy, college of medicine and health science, University of Gondar. All the study participants were informed about the objective of the study and their written consent was obtained. The confidentiality of the information was maintained.

### Operational definitions

**High diabetic health literacy:** If diabetic health literacy assessment score is 75% and above [10, 19].

**Moderate diabetic health literacy:** If diabetic health literacy assessment score is between 60–74% [10, 19].

**Low Diabetic health literacy:** If diabetic health literacy assessment score is less than 59% [10, 19]

**Good adherence:** 0 point from 4 item scale adherence assessment score [17, 18].

**Moderate Adherence:** 1–2 point from 4 item scale adherence assessment score [17, 18].

**Poor adherence:** 3–4 points from 4 item scale adherence assessment score [17] [18].

**Controlled glycemic target** is achieved when the FPG of the last follow up is between 80–130 mg/dl [20].

**Uncontrolled glycemic target** is when the FPG is either less than 80mg/dl or greater than 130mg/dl [20].

## Result

### Socio-demographic characteristics and clinical information of the diabetic patients

A total of 400 diabetic patients participated in this study. Slightly greater than half of the participants were females. Patients who had a family history of DM were accounted for half (50.3%) of the respondents and almost three fourth (76.5%) of the respondents represented the age group 40–60 years. Half of (51.2%) the respondents were on the oral hypoglycemic agents, and almost nearly half (45.8%) of respondents were on insulin plus oral hypoglycemic agent. The majority (61.8%) of the patients had no comorbidity associated with DM and nearly one fifth (19.5%) of the patients had hypertension. Most (81%) of the participants hadn’t developed any complication of type 2 DM. Neuropathy is the frequent (6.3%) diabetic complication experienced by the respondents. (**Table 1)**

**Table 1:** Socio-demographic characteristics and clinical information of the diabetic patients at the outpatient clinic of UOGCSH, 2019 (N = 400).

Variables		Frequency (%)
**Gender**	Male	189 (47.3)
	Female	211(52.8)
**Marital status**	Married	294(73.5)
	Single	35(8.8)
	divorced/widowed	71(17.8)
**Residency**	Urban	269(67.3)
	Rural	131(32.8)
**Educational status**	elementary school	82(20.5.)
	high school	106(26.5)
	higher institution	59(14.8)
	able to read and write	64(16)
	unable to read and write	89(22.3)
**Monthly income in ETB**	< 2000	95(23.8)
	2000–5000	198(49.5)
	5000–10000	83(20.8)
	>10000	24(6.0)
**Treatment regimen**	Diet/exercise only	1(0.3)
	Oral hypoglycemic agent	205(51.3)
	Insulin	11(2.8)
	Insulin + oral Hypoglycemic agent	183(45.8)
**Family history of DM**	Yes	201(50.3)
	No	199(49.8)
**Duration of disease**	Less than 5 years	155(38.8)
	5–10 years	155(38.8)
	Greater than 10 years	90(22.5)
**Age**	Less than 40 years	57(14.3)
	Between 40–60 years	306(76.5)
	Greater than 60 years	37(9.3)
**Occupational status**	Health professional	15(3.8)
	Government Employ	93(23.3)
	Merchant	126(31.5)
	Farmer	81(20.3)
	Housewife	53(13.3)
	Student	3(0.8)
	Others	29(7.3)
**Comorbidity**	None	247(61.8)
	Anemia	4(1.0)
	Arthritis	2(0.5)
	Chronic kidney disease	16(4.0)
	Chronic liver disease	2(0.5)
	Dyslipidemia	32(8)
	Heart failure	9(2.3)
	HIV	1(0.3)
	Hypertension	78(19.5)
	Hypertension + renal disease	1(0.3)
	Peptic ulcer disease	7(1.8)
	Stroke	1(0.3)
**Complication**	None	327(81.8)
	Coronary arterial disease	2(0.5)
	Diabetic foot ulcer	10(2.5)
	Nephropathy	19(4.8)
	Neuropathy	25(6.3)
	Peripheral arterial disease	4(1.0)
	Retinopathy	12(3.0)
	Stroke	1(0.3)
**Social drug history status**	None	129(32.3)
	Coffee	129(32.3)
	Tea	46(11.5)
	Alcohol	17(4.3)
	Cigarette	4(1.0)
	Chat	14(3.5)
	Tela/Tej	31(7.8)
	Coffee + Alcohol	29(7.3)

### Diabetic health literacy of the respondents

Two hundred twenty-six patients (56.5%) were considered to have high diabetic-related health literacy, 105 (26.3%) had moderate diabetic-related health literacy, and sixty-nine (17.3%) had low diabetic-related health literacy. Almost all participants either agreed or strongly agreed to the most questions except questions regarding determine the carbohydrate content per serving from the nutrition label and understand information on diabetes presented as probabilities, ratios, or on graphs which agreed or strongly agreed were 8.5% and 31%, respectively. In the questions that asses, the understanding of the information on diabetes management from the health-care provider was agreed or strongly agreed (78.3%), explanations on the diabetes condition to a healthcare provider was 79.3% and 73.8%for the knowledge and practice of the appropriate storage conditions of diabetic medications. More than three fourth (77.8%) of the respondents convey the reason for having a diabetic diet (**Table 2**).

**Table 2:** Diabetic health literacy of the diabetic patients at the outpatient clinic of UOGCSH, 2019 (N = 400).

Diabetic Health Literacy Questions	Strongly Disagree (%)	Disagree (%)	Neutral (%)	Agree (%)	Strongly agree (%)
Read and understand educational materials and booklets	52 (13%)	42(10.5%)	48(12.0%)	149(37.3%)	109(27.3%)
Understand the written information provided at the appointment	37 (9.3%)	49(12.3%)	33(8.3%)	182(45.5%)	99(24.8%)
Comprehend the information I sought on diabetes	41 (10.3%)	64 (16.0%)	43 (10.8%)	140 (35.0%)	112 (28.0%)
Understand the information on diabetes management from the health-care provider	11 (2.8%)	34 (8.5%)	42 (10.5%)	214 (53.5%)	99 (24.8%)
Judge if diabetes-related information is reliable	18 (4.5%)	61 (15.3%)	57 (14.3%)	151 (37.8%)	113 (28.3%)
Alter the appointment date or time for a medical checkup	57 (14.3%)	85 (21.3%)	54 (13.5%)	134 (33.5%)	70 (17.5%)
Calculate the next time to take diabetes medication	18 (4.5%)	64 (16.0%)	40 (10.0%)	179 (44.8%)	99 (24.8%)
Determine the carbohydrate content per serving from the nutrition label	193(48.3%)	134 (33.5%)	39 (9.8%)	22 (5.5%)	12 (3.0%)
Interpret if my blood-glucose level is within the normal range	50 (12.5%)	61 (15.3%)	47 (11.8%)	163 (40.8%)	79 (19.8%)
Understand information on diabetes presented as probabilities, ratios, or on graphs	40 (10.0%)	61 (15.3%)	171(42.8%)	52 (13.0%)	76 (19.0%)
Ask health professionals a question	18 (4.5%)	39 (9.8%)	38 (9.5%)	194 (48.5%)	111 (27.8%)
Explain my diabetes condition to a healthcare provider	19 (4.8%)	21 (5.3%)	43 (10.8%)	205 (51.3%)	112 (28.0%)
Convey the reason why I should have a diabetic diet	8 (2.0%)	28 (7.0%)	53 (13.3%)	195 (48.8%)	116 (29.0%)
Knowing and practicing the appropriate storage conditions of diabetic medications	6 (1.5%)	39 (9.8%)	60 (15.0%)	208 (52.0%)	87 (21.8%)
Understand all diabetic-related medication information	24 (6.0%)	76 (19.0%)	41 (10.3%)	179 (44.8%)	80 (20.0%)

### Mean score of diabetic health literacy

The overall mean diabetic health literacy score of the respondents was 3.68±0.82. Mean diabetic health literacy is higher in male, urban residents, patients with a family history of DM with P-value ≤0.001. One-way ANOVA also revealed there is a mean difference in diabetic health literacy score among patients with different educational status, occupational status and monthly income with P-value ≤0.001 (**Table 3**).

**Table 3:** Independent samples t-test and one-way ANOVA for mean diabetic health literacy score of the diabetic patients at the outpatient clinic of UOGCSH, 2019 (N = 400).

		Mean ± SD	F	Sig (P-value)
**Gender**	Male	3.8428 ±0.71194	11.663	≤0.001*
	Female	3.5414±0.87809		
**Residency**	Urban	4.0163±0.59317	19.976	≤0.001*
	Rural	3.0011±0.79013		
**Family history of DM**	YES	3.8496 ±0.73549	6.779	≤0.001*
	NO	3.5164±0.86158		
**Educational status**	elementary school	4.0240±0.59867	106.115	≤0.001*
	high school	4.0044±0.42972		
	higher institution	4.2518±0.48846		
	able to read and write	3.6561± 0.68709		
	unable to read and write	2.6320±0.64268		
**Occupational status**	Health professional	4.6127±0.42896	45.492	≤0.001*
	Government Employ	4.0461±0.53007		
	Merchant	3.9794±0.48228		
	Farmer	2.7885±0.70510		
	Housewife	3.4183±0.90176		
	Student	4.8667±0.11547		
	Others	3.6207±0.79164		
**Monthly income**	< 2000	3.2026±0.90677	18.465	≤0.001*
	2000–5000	3.8532±0.71169		
	5000–10000	3.8964±0.65054		
	>10000	3.4562±0.97400		

### Adherence level of diabetic patients

More than half (56.3%) and a third (34%) of the respondents had moderate and good adherence respectively while nearly one in ten (9.8%) of respondents had poor adherence level.

### Association of diabetic health literacy and adherence profile of the respondents

The majority (63.24%) of patients with good adherence level were those with a high diabetic health literacy level with P-value 0.023 while a nearly equivalent number of patients from lower and higher diabetic health literacy level had a lower adherence level **(Table 4).**

**Table 4:** Cross-tabulation and Pearson Chi-square test of diabetic health literacy level and adherence of the diabetic patients at the outpatient clinic of UOGCSH, 2019 (N = 400).

		Adherence Level				
		Good	Moderate	Poor	Total	P-Value
**Diabetic Health Literacy**	Low diabetic health literacy	25(18.38%)	33(14.6%)	11(28.2%)	69	0.023*
	Medium diabetic health literacy	25(18.38%)	68(30.2%)	12(30.7%)	105	
	High diabetic health literacy	86(63.24%)	124(55.1%)	16(41.1%)	226	
	**Total**	136	225	39	400	

### Factors associated with target glycemic achievement

Being in the age group of less than 40 years old is 6.23 times more likely to achieve the target glycemic control as compared with those older than 60 years with 95% CI AOR 6.23(1.99–9.11). Diabetic patients with morbidity have 67% less likelihood to achieve the target glycemic control; 95% CI AOR 0.33(0.15–0.73). Patients with High diabetes literacy are 1.85 times more likely to achieve target glycemic control when compared with lower health literacy level; 95% CI AOR 1.85(1.09–3.40). While patients with good adherence 1.61 times more likely to achieve target glycemic control than patients with low adherence; 95% CI AOR 1.61(1.04–4.79). (**Table 5**)

**Table 5:** Binary and multivariable logistic regression analysis for target glycemic achievement of diabetic patients at UOGCSH, 2019 (N = 400).

Variable	Target Glycemic Achievement (FPG = 80–130 mg/dl)		Crude Odds Ratio (COR) (95% CI)	P-Value	Adjusted Odds Ratio (AOR) (95% CI)	P-Value
	Controlled (%)	Uncontrolled (%)				
**Gender**						
Male	41 (21.69)	148 (78.31)	1.61(0.96–2.69)	0.07	1.71(0.87–3.37)	0.12
Female	31(14.69)	180(85.31)	1		1	
**Residence**						
Urban	59(21.93)	210(78.07)	2.55(1.34–4.84)	0.004	0.72(0.27–1.93)	0.519
Rural	13(5.62)	118(94.38)	1		1	
**Educational status**				0.001		0.108
Elementary school	21(25.61)	61(74.39)	5.78(2.07–16.19)	0.001	0.20(0.02–1.65)	0.135
High school	18(16.98)	88(83.02)	3.44(1.22–9.67)	0.019	0.20(0.02–1.63)	0.133
Higher institution	21(35.59)	38(64.41)	9.28(3.26–16.48)	0.001	0.45(0.05–3.86)	0.468
Able to read and write	7(10.94)	57(89.06)	2.06(0.62–6.82)	0.235	0.12(0.01–1.05)	0.056
Unable to read and write	5(5.62)	84(94.38)	1		1	
**Age**				0.001		0.045
Less than 40	22(38.6)	35(61.4)	11.00(2.4–20.37)	0.002	6.23(1.99–9.11)	0.049*
40–60	48(15.67)	258(84.33)	3.25(0.76–13.99)	0.113	2.52(0.47–3.61)	0.283
Greater than 60	2(5.41)	35(94.59)	1		1	
**Duration of the disease**				0.268		0.107
Less than 5 years	34(21.94)	121(78.06)	1		1	
5–10 years	24(15.48)	131(84.52)	0.65(0.37–1.16)	0.147	1.95(0.87–4.40)	0.106
Greater than 10 years	14(15.55)	76(84.45)	0.6(0.33–1.30)	0.227	2.86(0.99–8.23)	0.052
**Presence of Morbidity**						
Yes	16(10.46)	137(89.54)	0.40(0.22–0.72)	0.03	0.33(0.15–0.73)	0.006*
No	56(22.67)	191(77.33)	1		1	
**Presence of Complication**						
Yes	7(9.59)	66(90.41)	0.43(0.19–0.98)	0.044	0.77(0.26–2.34)	0.649
No	65(19.88)	262(80.12)	1		1	
**Diabetic health Literacy**				0.001		≤0.001*
Low Literacy	10(12.82)	68(87.18)	1		1	
Moderate Literacy	13(11.3)	102(88.7)	2.00(0.20–3.63)	0.552	1.11(0.45–2.54)	0.187
High Literacy	49(23.67)	158(76.33)	2.21(1.18–5.10)	0.001	1.85(1.09–3.40)	≤0.001*
**Adherence Level**				0.001		≤0.001*
Good adherence	47(34.56)	89(65.44)	2.05(1.37–4.81)	0.01	1.61(1.04–4.79)	0.04*
Intermediate adherence	17(7.55)	208(92.45)	0.32(0.13–0.8)	0.014	0.19(0.06–0.59)	0.14
Poor adherence	8(20.51)	31(79.49)	1		1	

*P-value less than 0.05 CI -confidence Interval

## Discussion

Diabetic health literacy affects patient self-care behavior, medication adherence, health-seeking behavior which can contribute to poor glycemic control and disease progression. Target glycemic control is an achieving Fasting Plasma Glucose (FPG) level of 80–130 mg/dl or HbA1C less than 7% as per the American Diabetic Association. Achieving FPG between 80–130 mg/dl is less pragmatic glycemic control classification to various patient populations with various morbidities, complications, estimated short life expectancy and longer duration of the disease. But, it could be an alternative for patients' glycemic control as a stringent parameter for patients and health facilities lacking HbA1C which is an ideal predictor of glycemic control for the last three months [20, 21]. The main needed outcome in diabetes management is achieving glycemic control and prevent or delay complications associated with diabetes. This can be mainly achieved through having good diabetic literacy, adherence to the medication and other associated self-care management [22]. No prior study conducted in Ethiopia regarding diabetic health literacy using the diabetic health literacy scale and its association with medication adherence and its impact on glycemic control. This study assessed the glycemic status as determined by FPG, and factors associated with good glycemic control among patients with T2DM. FPG is more acceptable than Random Blood Glucose (RBG) since the result would not fluctuate or affected by the prandial (meal) as that of RBG.

In the present study, most patients disagreed to the question asking regarding determining the carbohydrate content from their nutrition. This is due to the nutrient label is not known in developing countries food customs since nutrition labeling is common for packed and processed foods which are widely used only in developed countries [23]. The overall mean diabetic health literacy score in this study was 3.68 out of 5-point Likert scale which is higher than the study done in Palestine patients mean health literacy of 2.9 [22]. This might attributable to the variability of health literacy tools used since the 4-item health literacy tool used in the later study. But also, the sociocultural and geographical variation might explain further differences. The majority of diabetic patients who account for more than half had a good diabetic health literacy level. This finding is higher than Tefera et al study in Southwestern Ethiopia which is slightly more than one-third had good diabetic related knowledge [19]. This might be due to the continuous morning diabetic health education provided for patients who are under regular follow-up. Besides this, in the present study setting a relatively higher number of medical practitioners are available and patients might have “adequate” consultation time with doctors and likely to be receiving better diabetic care and support. Adequate health literacy and a better understanding of health education are highly correlated. The association of adequate health literacy in achieving good glycemic control may not be genuine in patients with a better-perceived understanding of health education and instructions. There is often overestimation of high health literacy since some patients who have inadequate health literacy skills often deny or conceal their deficit. Healthcare providers can facilitate informed decision-making and facilitate action to improve personal capacity to exert control over factors that determine health and improve diabetic outcomes [24]. Consistent diabetic education should be encouraged to increase patient diabetic health literacy and medication adherence as both are the most cost-effective prevention and management strategies for DM complications [25].

In the present study, higher diabetic health literacy is seen in male which is similar to finding in Kamuhabwa et al study conducted in Tanzania [26]. This might also potentially explained to women generals’ literacy index is lower than men for various gender-specific socio-cultural barriers [27]. In Ethiopia like most developing nations, women are usually do not have the same access to modern education and information as men do, this could affect the health literacy of women [27]. But no difference with males to achieve the targeted glycemic level. But different results reported in the studies conducted in the Gaza Strip, Palestine [22] and Japan [28]. This indicates differences in gender could not be explained by dissimilarities in body composition and therefore, there is a need for further investigation to examine sex-related distinction in efficacy/treatment response.

The mean score for diabetic health literacy is higher for those attending higher education and earning the highest monthly income. This is in agreement with a study conducted in China where higher education attainment and higher household income were significantly associated with adequate health literacy [9]. Diabetic patients who live in urban had a better diabetic health literacy level than those living in rural. This might be due to the various exposures in urban that could increase diabetic health awareness through audiovisual communications such as radio, television, computer, mobile technologies, and internet access. Patients with a family history of DM had also higher diabetic health literacy than patients without a family history of DM. This would be explained exposure to family’s diabetic history could increase disease awareness as compared with naïve patients. In one study having the family history of DM had an association with patient perception of understanding health education and instructions provided by their clinician and also found to be related to better self-care management [29]. It would be plausible to argue that diabetic patients would share their knowledge, and experiences with family members [30].

In the current study, the respondents younger than 40 years, is more likely to achieve the target glycemic control than older patients who are above 60 years. Younger patients were less likely to have low diabetic knowledge than those in the age group of older than 60 years. This indicates that older people were at increased risk and thus there would be a need to develop special education to address the disparity that existed in older age groups [19]. This is might be in the age group (<40 years) the attention they pay for their disease is good. The variation of the relationship between poor glycemic control and age could be due to the dissimilarities of the studied population and age distribution in various studies [19, 22]. One study found that during treatment of diabetes, if doctors behave in a way that makes old patients feel inferior, for example, ordering the patients to follow their instructions without allowing them to express any feelings or opinions, the doctors may not be able to receive sufficiently useful information from the patients, may not receive good participation from the patients, or the patients may decide to receive a treatment with a new doctor or at other hospitals [31]. Some patients feel that they do not receive good respect from medical staff; some feel irritated and annoyed; some feel that they are not well cared for by medical staff. These influence them to seek out new doctors, new hospitals or other alternative treatments such as herbal treatment or traditional treatment. This may cause the diabetic condition to get worse and poor glycemic control [32].

A significantly low diabetic health literacy level reported in illiterates than those who attained a higher level of education. This finding was consistent with other studies from the United Arab Emirates (UAE) [33] and Bangladesh [34] and southwest Ethiopia [19]. Unable to have access to formal education could end up with lower general literacy which might affect negatively the diabetic health literacy and self-care behaviors.

In the binary and multivariable logistic regression analysis, having diabetic related morbidity is a negative factor associated with poor glycemic control. Diabetic patients with morbidity have 67% less likelihood to achieve the target glycemic control. The glycemic target is individualized in thus patients with comorbidity since it is depending on the condition of the patient. But in this study, the ADA cut point used which was originally for an adult, non-pregnant and without any comorbid disease. Therefore, it might also overestimate the poor glycemic control of diabetes patients with morbidity [20].

Patients with high diabetes literacy have more likelihood to achieve target glycemic control than those with low diabetic health literacy. Almost those patients are expected to be aware of the disease state, management, and diabetic specific self-care. Better diabetic health literacy was significantly associated with good glycemic control. This is consistent with findings obtained in Kasper et al cohort study of Type 1 DM [35] and Cavanaugh et al study [36]. Knowledge has a significant contribution to self-care behaviors and medication adherence to achieve a target glycemic control [37]. As such, these findings inform the necessity to have consistent diabetic education to address issues related to diabetic health literacy and adherence to medications as both are the most feasible strategies for diabetes management [25]

Adequate glycemic control needs patients to know how to manage symptoms, monitor medical treatments daily, and self-monitoring of disease progression [22]. Therefore, inadequate health literacy hinders these practices, leading to irrational diabetes management and early occurrence of diabetic complications such as peripheral neuropathy, renal and ophthalmic complications [36]. High adherence to anti-diabetic medications was suboptimal (34%), which is comparable with the study conducted in Southwest Ethiopia [19], America [38] & [39] Tanzania [26], India [40] and Palestine [22]. In current study patients who have good adherence are more likely to achieve their glycemic control than those with low adherence. This is apparent to see good diabetic treatment outcomes when the patients adhered to their medication.

### Limitation of the study

HbA1c was not used, a more accurate indicator of glycemic control than FPG measurement due to inaccessibility in the study setting and most public hospitals of the country. Using the latest fasting blood sugar as an indicator of glycemic control is clinically less reliable as compared with HbA1C to predict glycemic control in diabetic patients. Target glycemic control is an achieving fasting plasma glucose level of 80–130 mg/dl as per the American diabetic association which might not be pragmatic to various patient populations since it is a very stringent classification that disregards specific patient clinical characteristics such as the presence of morbidity, complication, and duration of the disease. The study design it applied; as cross-sectional studies are poor in establishing the temporal relationship between cause and effect and the fact that it was conducted among the outpatients in only one hospital could limit our understanding regarding diabetic-related health literacy and its impact on glycemic control among general diabetic population.

## Conclusion

The current study revealed only the average number of diabetic populations have a good diabetic health literacy level. High diabetic health literacy reported in males, urban residents, patients who attended higher education and with a family history of diabetes. The majority of patients with good adherence level were associated with high diabetic health literacy level. Adjusting all variables; younger age, high diabetic health literacy and good adherence were associated with achieving the target glycemic control. Different patient empowerment programs and approaches aimed at raising diabetic health literacy would be essential to improve medication adherence and glycemic control.

## Supporting information

S1 Dataset (SPV)

## Abbreviations and acronyms

ADA: American Diabetic Association
CDC: Centers for Disease Control and Prevention
CI: Confidence Interval
DM: Diabetes Mellitus
ETB: Ethiopian Birr
FPG: Fasting Plasma Glucose
RBG: Random Blood Glucose
T1DM: Type 1 Diabetes Mellitus
T2DM: Type 2 Diabetes Mellitus
UAE: United Arab Emirates
UKPDS: United Kingdom Prospective Diabetes Study
UOGCSH: University of Gondar Comprehensive Specialized Hospital
WHO: World Health Organization
